# Differences in gene expression in prostate cancer, normal appearing prostate tissue adjacent to cancer and prostate tissue from cancer free organ donors

**DOI:** 10.1186/1471-2407-5-45

**Published:** 2005-05-13

**Authors:** Uma R Chandran, Rajiv Dhir, Changqing Ma, George Michalopoulos, Michael Becich, John Gilbertson

**Affiliations:** 1 From the Department of Pathology, University of Pittsburgh, Pittsburgh, PA 15232, USA

## Abstract

**Background:**

Typical high throughput microarrays experiments compare gene expression across two specimen classes – an experimental class and baseline (or comparison) class. The choice of specimen classes is a major factor in the differential gene expression patterns revealed by these experiments. In most studies of prostate cancer, histologically malignant tissue is chosen as the experimental class while normal appearing prostate tissue adjacent to the tumor (adjacent normal) is chosen as the baseline against which comparison is made. However, normal appearing prostate tissue from tumor free organ donors represents an alterative source of baseline tissue for differential expression studies.

**Methods:**

To examine the effect of using donor normal tissue as opposed to adjacent normal tissue as a baseline for prostate cancer expression studies, we compared, using oligonucleotide microarrays, the expression profiles of primary prostate cancer (tumor), adjacent normal tissue and normal tissue from tumor free donors.

**Results:**

Statistical analysis using Significance Analysis of Microarrays (SAM) demonstrates the presence of unique gene expression profiles for each of these specimen classes. The tumor v donor expression profile was more extensive that the tumor v adjacent normal profile. The differentially expressed gene lists from tumor v donor, tumor v adjacent normal and adjacent normal v donor comparisons were examined to identify regulated genes. When donors were used as the baseline, similar genes are highly regulated in both tumor and adjacent normal tissue. Significantly, both tumor and adjacent normal tissue exhibit significant up regulation of proliferation related genes including transcription factors, signal transducers and growth regulators compared to donor tissue. These genes were not picked up in a direct comparison of tumor and adjacent normal tissues.

**Conclusions:**

The up-regulation of these gene types in both tissue types is an unexpected finding and suggests that normal appearing prostate tissue can undergo genetic changes in response to or in expectation of morphologic cancer. A possible field effect surrounding prostate cancers and the implications of these findings for characterizing gene expression changes in prostate tumors are discussed.

## Background

Prostate cancer is the most common cancer in men resulting in over 30,000 deaths annually [1]. Early detection and treatment has the potential to markedly reduce the morbidity and mortality associated with the disease. While elevated Prostate Specific Antigen (PSA) [2] is the best available indicator of men with cancer [3], its diagnostic utility is limited due to elevated PSA levels in other non-malignant prostate conditions, varying levels in advanced disease and poor correlation between PSA levels and extent of disease. Furthermore, the variable course of prostate cancer – many patients will not die of the disease – means that radical therapy for all early cases would result in over treatment of significant number of patients. High throughput genomic technologies, by simultaneously interrogating the expression levels of thousands of genes, offers the potential to identify new biomarkers for early detection, prognosis, targets for therapy and for reclassification of prostate tumors. Using expression microarrays, a number of studies have characterized expression profiles for prostate cancer and other tumors. In some cases, correlations between tumor expression signatures, clinical parameters and outcome [4-12] have been identified. While potentially powerful, such studies can be significantly impacted by the choice of baseline or "normal" tissue used to detect tumor related expression changes. Most prostate expression studies to date have utilized normal appearing tissue adjacent to tumor as the tissue for comparison. However, a variety of methods such as chromosomal analysis [13], SAGE [14] and ploidy analysis [15,16] have shown molecular abnormalities in normal appearing prostate adjacent to tumor. Even the term "normal appearing" prostate tissue adjacent to tumor may be misleading, as morphologic researchers using quantitative imaging analysis [17-19], have identified morphologic changes in the epithelial nuclei and blood vessels architecture in prostate tissue adjacent to tumor that are not routinely commented upon by pathologists. This suggests that, in some cases, tissues adjacent to cancer, although appearing morphologically normal by traditional microscopic examination, may contain genetic changes associated with the genesis of or reaction to cancer. Therefore, the use of adjacent normal as the baseline tissue for comparative gene expression studies may mask tumor related molecular changes preceding the appearance of histological tumor. More recently, a microarray study from our institution [12], using adjacent normal and tumor samples, describes a potential field effect around prostate cancers and regulation of selected genes in both adjacent normals and tumors. Using the same microarray data set for our analysis, we have compared the gene expression profiles of prostate cancer, normal appearing prostate tissue adjacent to tumor, and normal appearing prostate tissue from cancer free tissue donors with the aim of identifying the optimal baseline tissue for expression studies and the gene expression changes between the three specimen types.

## Methods

### Clinical profile of cases

The 60 tumor samples used in this study consisted of 2, 13, 27, 6 and 12 cases of primary prostatic adenocarcinoma of Gleason grade 5, 6, 7, 8 and 9 respectively. There were 4, 20, 23 and 13 cases spanning the age groups 40–49, 50–59, 60–69 and 70–79 respectively. Of the cases, 36 were stage T3 or higher with 2, 22, 23, 11 and 2 cases of stage T2a, T2b, T3a, T3b and 4 respectively.

The 63 adjacent normal samples consisted of 2, 11, 29, 8, and 13 cases of Gleason grade 5, 6, 7, 8 and 9 respectively. There were 4, 21, 25 and 12 cases spanning the age groups 40–49, 50–59, 60–69 and 70–79 respectively. There were 2, 21, 26, 12 and 2 cases of Stage T2a, T2b, T3a, T3b and T4 respectively.

Of the donors, 11 are under and 7 are over the age of 40.

### Samples and sample procurement

The tumor and adjacent normal tissue samples were acquired from the University of Pittsburgh Medical Center under stringent Institutional Review Board guidelines with appropriate informed consent. Specimens were received directly from the operating room. Samples (>500 mg) were excised and snap frozen in liquid nitrogen within 30 min of excision and stored at -80°C in the University of Pittsburgh Pathology Tissue Bank until extraction of RNA. All samples were submitted for pathology evaluation. In every case, the tissue was excised from the junction between the ejaculatory duct and the prostatic urethra in the transition zone of the prostate. In particular, adjacent normal tissue was excised away from the cancer lesion macroscopically, and their histological diagnosis was confirmed microscopically.

Donor tissue specimens were received through a collaborative arrangement with the Center for Organ Recovery and Education (CORE), the local organ procurement agency. The arrangement allows the University of Pittsburgh Pathology Tissue Bank to acquire normal prostates and associated serum/plasma specimens from healthy individuals who have donated their organs for transplant. There is extensive collaborative support from CORE. The donor prostatectomies harvested from brain dead, perfused donors and are bathed in Ringer's Lactate solution and transported on wet ice. These donor prostates are transported and handled with the harvested "transplant" organs. This significantly reduces transit time and minimizes the degradation of RNA. The processing methodologies used consist of snap freezing tissues in bulk, freezing in OCT and processing the tissues for routine histology (paraffin embedded tissues. For microarray analysis, the donor samples were excised from the same zone as the tumor and adjacent normal samples.

### cRNA preparation and Affymetrix chip hybridization

cRNA was prepared and hybridized to Affymetrix oligonucleotide arrays as previously described [12]

### Statistical analysis

We analyzed prostate tissue samples from 18 donors and 63 prostate cancer patients. From the prostate cancer patients we took samples from the histologic tumor as well as normal appearing tissue adjacent to the tumor. High quality RNA and chip data were obtained from 60 cancer and 63 adjacent to tumor samples. In total 141 samples were run against the Affymetrix U95A chip and analyzed. The raw scanned array images were first processed through the Affymetrix Microarray Analysis Suite 5.0 (Affymetrix Corporation, Santa Clara, Ca) to generate probe cel intensity (*.cel) files. The *.cel files were then analyzed using both MAS 5.0 and dChip software from Harvard University [20], to generate gene expression signal values for each probe set. Data normalization to remove variation in overall chip intensities was perfumed by global scaling to a chip mean target intensity of 200 (MAS 5.0) or by the rank-invariant method (dCHIP). The MAS 5.0 with global scaling data and dChip with rank-invariant normalization data gave similar results in the subsequent analysis. Therefore, in the interests of clarity, we will focus on the MAS 5.0 results in the remainder this paper.

In the next phase of analysis, the donors, adjacent normals and tumors were compared for differences in gene expression by using signal values for all 12625 probe sets for each sample. For statistical analysis, we used the Significance Analysis of Microarray (SAM) software package from Stanford University [21]. This method was chosen over conventional statistical tests because of its acceptance in the microarray community, its general simplicity and its ability to provide an estimate of the false discovery rate (the ratio of false positives to total positives). The false discovery rate is particularly important when comparing the expression of thousands of genes simultaneously. For example, when using the Student's t test at a P value of 0.05 to examine a population of 10,000 genes, one would expect 500 false positives. If there were in fact 100 true positives, the false positive rate would be an unacceptably high 0.83 (500/600).

Briefly, SAM calculates a value for each probe set on the array. This value represents the observed difference in mean expression levels between the specimen classes being compared (i.e. tumor and donor) divided by the variance in the data and a fudge factor (see the original paper for details [21]. The resulting value is called the "observed d value". To determine the significance of this value, SAM estimates the "expected" d value if there were no difference between the specimen classes. This is done by permutating (randomly changing) the class labels without changing the data and recalculating the SAM value for each probe set. After thousands of permutations, the result estimates the value that would be obtained if the difference in gene expression were due to chance alone. This is the "expected d value".

The significance of the observed differential gene expression can be estimated by comparing the observed and expected d values. A user defined threshold or "delta" (observed d value – expected d value) can be adjusted to select only those genes observed d value exceeds (for up regulated genes) or is lower (for down regulated genes) than delta. The greater the "delta", the greater the stringency of the result and lower the false discovery rate. For each delta value, the SAM output consists of a gene (probe set) list and an associated false discovery rate. The false discovery rate is estimated from the distribution of expected and observed d values. Probe sets are ordered on the basis of observed d value metric, probe sets with high (or low) values represent genes with relatively high differential expression. The "SAM Plots" are also very useful in visualizing differences in overall differential gene expression between specimen classes.

## Results

### Expression analysis of tumor, adjacent normal and donor tissue

The prostate tumors analyzed in this study consisted of Gleason grades 5, 6, 7, 8, 9 and patients spanned the ages of 40 through 79. The goal of our research was to examine the differential gene expression patterns observed when comparing our three specimen classes: tumor versus adjacent normal, tumor versus donor and adjacent normal versus donor. The comparison was made at three points in the analytical process: 1) after normalization to remove variation in overall chip intensity 2) after statistical analysis of the data and, 3) after examining the differentially expressed gene lists.

**Figure 1:**
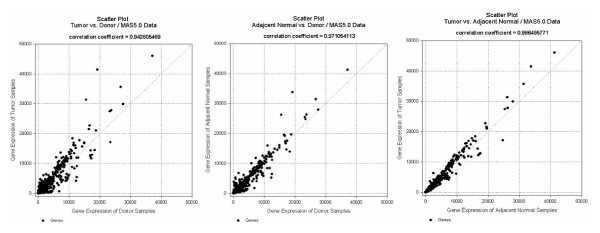
**Differential gene expression analysis of donor, tumor and adjacent normal protate cancer samples. **Scatter plot of MAS 5.0 derived tumor vs donor, adjacent normal v donor and tumor v adjacent normal samples. For each probe set, the mean MAS 5.0 expression values of all the samples in each specimen group was calculated. Scatter plot were constructed using the mean values for each specimen group.

To examine differences in normalized gene expression between tumors, adjacent normals and donors the mean MAS 5.0 and dChip generated signal of each probe set for each specimen class (60 tumors, 63 adjacent normals and 19 donors), was calculated and plotted on a series of scatter plots (Fig 1).

Figure 1 shows the scatter plots and Pearson correlations of the normalized expression data analyzed using both MAS 5.0 and dChip as described above (vide supra, Methods). Data scatter is maximum in the tumor versus donor comparison, intermediate in adjacent normal versus donor and minimal in tumor versus adjacent normal. These findings are suggestive of more differential gene expression in tumor versus donor than tumor versus adjacent normal. In other words, donor normal tissue and adjacent normal tissue do not show the same degree of differential gene expression when paired with tumor tissue. Another striking result apparent in Figure 1 is the close correlation and limited scatter of the tumor versus adjacent plot, even at low levels of signal.

**Figure 2:**
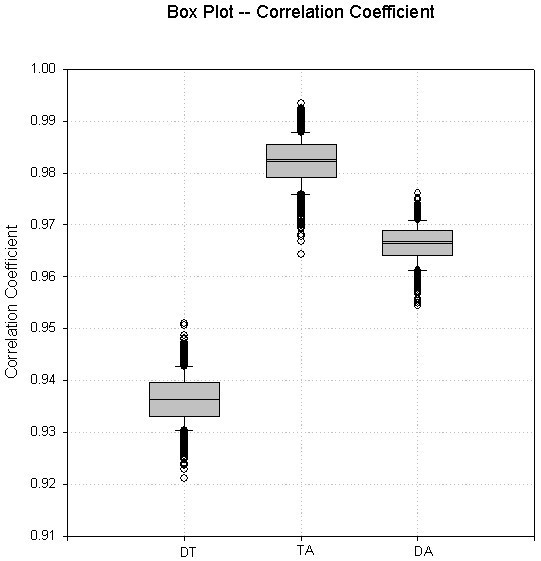
**Regression analysis of permuted donors, adjacent normal and tumor samples. **The 60 tumors and 63 adjacent normal tissues were segmented so that tumors and adjacent normal samples in each comparison were selected from different patients. The resulting tumor and adjacent normal samples were then subjected to regression analysis. Donor v tumor, donor v adjacent normal and adjacent normal comparisons were performed. Since the segmentation can be performed in many different ways, the analysis was performed 1000 times. The mean correlation coeifficient and standard deviation from each of these comparisons were plotted as box plots.

Tumor and adjacent normal specimens came from the same population of patients while donor specimens were received from a different set of individuals. To examine potential patient specific expression effects, the 60 tumors and 63 adjacent normal cases were randomly segmented into two groups, one group provided just tumor data and the other just adjacent normal data and a scatter plot of expression was generated. Since the segmentation of 63 cases can be performed in many different ways (permutations), the scatter analysis was performed 1000 times and the correlation between the sample groups determined by obtaining the mean correlation coefficient of the 1000 permutations. (Figure 2). In this analysis, the close correlation in expression between tumor and adjacent normal specimens persisted even when tumor and adjacent normal samples were taken from different patients.

**Figure 3:**
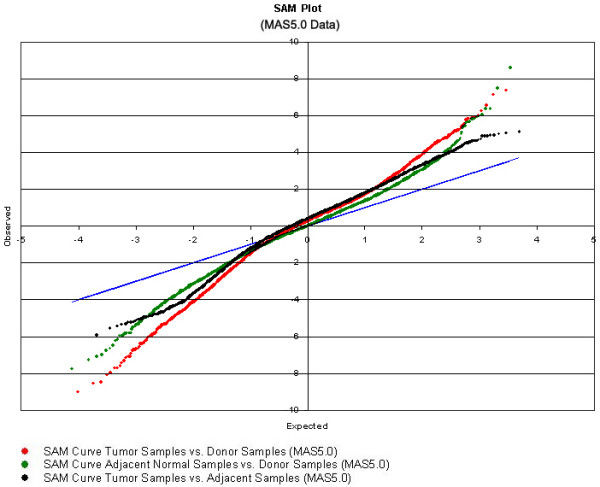
**Overlayed SAM plots (for details, see Materials and Methods) from the donor v tumor, donor v adjacent normal and tumor v adjacent normal analyses. **Each of the SAM plots was overlayed to direct comparison of the plots. The diagonal line represents no differential gene expression where the observed d value equals the expected d value after 1000 permutations of the class labels. Genes that are differentially expressed are displaced from the diagonal (greater than 0 for up regulation and less than 0 for down regulation). Genes that are more differentially expressed are more displaced from the diagonal than those that are closer to the diagonal. For each of the comparisons, a plot is generated from the d values of the 12625 probe sets in the two specimen groups. Red = donor v tumor plot; green = adjacent normal v tumor plot; black = adjacent normal v donor plot.

To determine the statistical significance of the observed differential expression between the three specimen groups, SAM analysis was performed. From each comparison (tumor v adjacent normal, tumor v donor and adjacent normal v donor), a SAM plot was generated and the plots for the three comparisons were overlaid (Fig 3). The diagonal line in Figure 3 represents no differential expression (identical observed and expected d values, for further details see Materials and Methods) with points displaced from the diagonal representing differential expression. Figure 3 shows that each of the comparisons yields a distinct expression profile with donor v tumor exhibiting more differential expression than adjacent normal v tumor or donor v adjacent normal.

To further characterize the expression profiles from these comparisons, differentially expressed gene lists were created from each comparison by selecting genes whose d values (for details, see Materials and Methods) exceed a given threshold. False discovery rates (false positives/ total number of genes in gene list) is no greater than 2.5% at the deltas chosen for this analysis (Table 1).

At a delta of 2.0, when tumor expression is compared to donor expression (Table 1), 474 differentially regulated genes can be detected. At the same delta, when tumor expression is compared to adjacent normals, only 92 genes are differentially regulated between these two tissues. Furthermore, at this delta, comparison of tumor expression with adjacent normals does not yield any genes up-regulated in tumors whereas the comparison with donors demonstrates up-regulation of 121 genes. Similarly at other deltas, approximately three times more differentially regulated genes can be detected when tumors are compared to donors than to adjacent normals.

As was discussed above, tumors and adjacent normal tissues are obtained from the same patients and donor tissues from a different sample population. Therefore the larger gene expression differences between tumors and donors may represent underlying patient specific (genetic, demographic or handling) differences in patient (tumor and adjacent normal) and donor prostates rather than intrinsic differences between tumor, adjacent normal and donor normal tissues. It is significant however, that SAM analysis indicates that adjacent normals exhibit far less differential regulation than tumors when both are compared to donors. At all deltas (Table 1), tumors v donors exhibit greater differential expression than adjacent normals v donor implying that tumors and adjacent normals are not identical in gene expression. Therefore, tumor specific, and not patient specific, expression changes can indeed by detected by comparing tumors to donor prostates. Significantly, these results establish the presence of unique gene expression profiles for prostate tissue from donors, adjacent normals and tumors (see Fig 3) with tumors differing more from donors than from adjacent normals.

A potential limitation of our data is that donors span the ages of 5 to 60 and all tumor patients are older than 40. Therefore, the differential gene expression between donors and patients may be due to age specific differences in their prostates. To examine this, we segmented the donors into different age groups and compared only the 40 to 60 year old donors with tumors of the same age group. Although the number of cases in the study were small, the expression pattern observed in this age matched analysis is identical (data not shown) to that when all donors are included suggesting that potential age related differences in donor prostates do not contribute to the results of the donor v tumor analysis.

### GO annotation of differential gene expression

We examined the gene lists produced by SAM analysis of tumor, adjacent normal and donor tissue with two objectives: 1) to identify and functionally annotate some of the genes that contribute to the unique expression signatures of these tissues and 2) to determine whether adjacent normals or donors are the more appropriate baseline tissue for detecting differentially expressed genes in tumors. Functional annotation and comparison of the gene lists was performed using Gene Ontology terms [22], for biological processes and Affymetrix's Gene Ontology Mining Tool .

When donors are used as the baseline for comparison, tumors exhibit up-regulation of proliferation related genes including transcription factors, signal transducers and growth regulators (see additional file 1). This list includes putative oncogenes, signal transducers and growth regulators. Some of the most up-regulated genes are *v-fos*, *jun B*, *jun D*, *c-src tyrosine kinase*, *FGF receptor activating protein, immediate early protein *and *early growth response 1*. The most down-regulated genes in tumors include those involved in immune response and signal transduction. Some of the genes in this list are the *interferon induced transmembrane proteins, Duffy blood group antigen *and *tumor necrosis factor*. In contrast, when adjacent normal tissue is used as the baseline for comparison, tumor tissue exhibits far fewer differentially expressed genes and the genes themselves are less compelling. The list up regulated genes is dominated by ribosomal proteins and metabolic enzymes, while the down-regulated list includes muscle related genes such as *tropomysin*, *actin *and *actinin*.

When expression in adjacent normal is compared to donors, an up-regulation pattern remarkably similar to tumors is seen (additional file 1). Adjacent normals also exhibit up-regulation of putative oncogenes, signal transducers and growth regulators with an almost 70% overlap of the 50 and 100 most up-regulated genes in tumors and adjacent normals, respectively. Similarly there is almost 60% overlap between the most down-regulated genes in tumors and adjacent normals that includes genes involved in immune response.

**Figure 4:**
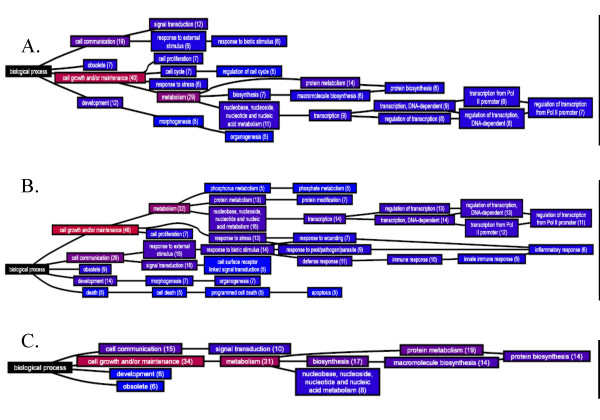
**Gene Ontology annotation of differentially expressed gene lists. **The fifty most upregulated genes from the donor v tumor, adjacent normal v tumor and tumor v adjacent normal comparisons were uploaded to Affymetrix's Gene Ontology Mining Tool, *a*, donor v tumor; *b*, donor v adjacent normal; *c *adjacent normal v tumor; The annotations is presented as a hierarchy of terms, from general to most specific terms (from left to right). The numbers in parenthesis indicate the number of genes that are annotated with the term. In all of the analysis, annotation of all the submitted probe sets is not achieved. Typically, annotation exists for approximately 60% of the probe sets.

The biological processes regulated in tumors and adjacent normals were also studied using Affymetrix's Gene Ontology Mining tool. The up regulated gene lists obtained at a SAM delta of 2.0 (Table 1) were uploaded to the tool and the resulting annotations examined. Comparison of tumor gene expression to donor expression reveals up-regulation of genes involved in a number of biological processes (Figure 4a). Amongst these are genes involved in apoptosis, cell cycle, cell proliferation, immune response, protein phosphorylation, protein biosynthesis and transcription. A subset of these including genes involved in immune response and transcription are also up-regulated in adjacent normals (Figure 4b). In contrast when tumor expression is compared to adjacent normals, up-regulation of majority of these processes, except protein metabolism, is not detected (Figure 4c).

Two important conclusions can be derived from the gene annotations, 1) though there are large number of genes regulated in tumors, there is a relatively small subset of genes including oncogenes and signal transducers that are highly regulated in both adjacent normal and tumor tissues and 2) regulation of a number of potentially important biological processes in tumors can be detected from using donors as the baseline tissues. The common regulation of oncogenes, signal transducers and immune response genes in adjacent normals is a striking result in that it suggests that adjacent normal tissue although appearing morphologically normal, undergo gene expression changes that may be important in tumorigenesis or as a reaction to tumor. Since these genes are regulated in both tumor and adjacent normal, they are not picked up on a direct comparison of the two tissues. While it is possible that donors are different from both adjacent normals and tumors due to processing artifacts – the tumor and adjacent tissues were taken at surgery and donors at harvesting – it is unlikely that the large differences seen in donor v tumor are all due to processing differences. This issue is examined further in the discussion section.

The up regulation of proliferation markers in both adjacent normals and tumors coupled with the result that more differential regulation is detected when tumors are compared to donors than to adjacent normals suggests that donor prostates may be the more appropriate tissue for expression studies. Regulation of critical of biological processes and pathways may remain undetected if tissue adjacent to tumors is used for comparison.

## Discussion

There is a growing interest in the use of high throughput microarray analysis for the molecular reclassification of diseases. This interest appears to be well founded, as many groups have reported consistent patterns of gene expression associated with pathologic phenotypes, clinical behaviors and outcomes [4-11]. In the area of prostate cancer numerous groups [23-29] have all reported significant differential gene expression between histologic tumor specimens and normal appearing prostate tissue from patients with tumor present elsewhere in the prostate. Recently, a group from our institution reported a 70 gene signature that may predict aggressiveness of prostate cancer [12]. Comparison of the gene lists from published data sets with the results of our tumor versus adjacent normal analysis is complicated by the heterogeneity in samples, analysis platforms and analysis methods. Nevertheless, our study is qualitatively similar to other studies in the expression profile of tumors compared to adjacent normal tissue. A number of genes including *hepsin*, *myc*, *fatty acid synthase SPARC1 *and *EBNA-2 *coactivator show similar expression patterns across multiple prostate cancer studies [30], and are also regulated in our study.

Our donors did not have prostate cancer or prostatic intraepithelial neoplasia (PIN) identified in their prostate and as such are good candidates for "true normals". Differential expression was much greater between tumor and donor tissue than between tumor and adjacent normal. The fact that tumor and adjacent specimens come from the same patients could possibly explain this difference but this was ruled out by our analysis. Another possibility is that tissue handing and processing differences could account for some or all of the differential expression seen when donor tissue is use as a baseline. In fact, data in the literature does suggest that tissue processing could effect the expression of genes such as *fos*, *jun *and *egr *in prostate tissue [29]. However, the same literature indicates that the effect warm ischemic time is limited to specific genes and in general, involves less than 1% of the regulated genes [29]. Our studies emphasize the need for documentation and quality of all experimental processing steps, from sample acquisition to sample hybridization, in order to completely characterize gene expression differences between prostate donors, tumors and normal tissue adjacent to tumors.

In our experiment, tumor and adjacent normal specimens where taken from the same prostates and handled the same way. If differences in patient and donor tissue handling was the major issue driving differential expression in the tumor v donor and adjacent normal v donor comparisons, one would expect tumor v donor and adjacent normal to result in very similar expression profiles. However, we have shown that tumor v donor exhibits far greater differential expression than adjacent normal v donor (see Results). Furthermore, the differentially expressed genes seen in both tumor and adjacent normal include proto-oncogene and transcription factors that one might rationally expect to see in expectation of or in response to a local tumor. Therefore, while the possibility that some expression changes are due to differences in tissue handling cannot be formally ruled out, it is unlikely that the large and specific differences we observe in tumor v donor, tumor v adjacent normal and adjacent normal v donor are entirely due to processing differences. Clearly additional studies, including examination of patient process specimens that do not host prostate cancer (such as cystoprostatectomy for bladder cancer or prostates removed for benign hypertrophy) to examine this process further.

The most important finding from our analysis is the potential importance of the donor specimens and the possibility that a field effect exists around prostate tumors, resulting in significant molecular changes in histologically normal appearing tissue adjacent to prostate cancer. Significantly, evidence for such malignancy associated changes have been presented in other organs such as the cervix, bladder and breast [31-33]

Furthermore, a variety of methods such as chromosomal analysis [13], SAGE [14], ploidy analysis [15,16] have shown molecular abnormalities in normal appearing prostate adjacent to tumor. Image analysis has also been employed to identify consistent changes in "normal appearing" prostate tissue adjacent to tumor [17,18]. In one study cases of prostatic adenocarcinoma was consistently detected by examining histologically normal tissue using high-resolution image cytometry [18], and in another, combined highly sensitive and discriminating Fourier transform-infrared spectroscopy with statistical analysis was used to detect damaged DNA in normal appearing prostate tissue adjacent to cancer [34].

In expression analysis, while most published prostate studies have used adjacent normals as the baseline tissue, Dhansekaran [23], used both commercially available pooled donor normal tissue and adjacent normal tissue and noted differences in expression profile between the two specimen types. Genes that were differentially expressed in adjacent normals when compared to the pooled donor normals included signal transducers and transcription factors; and expression of these genes in adjacent normals was attributed to a field effect around tumors. Similarly, Yu [12] have noted dysregulation of selected genes in both adjacent normals and tumors when compared to donors. Prakash [27], found that gene expression in asymptomatic benign prostatic hyperplasia adjacent to tumors was different from asymptomatic BPH or symptomatic BPH not associated with tumors. The unique expression signature of BPH next to tumors included fos, jun, immediate early genes and this list was remarkably similar to the most up-regulated genes in the adjacent normals tissue in our study (see adjacent normal v donor, additional file 1).

Finally, within archives of the University of Pittsburgh Pathology Tissue Bank, there was a donor prostate, which was found to harbor prostate cancer. When run on the Affymetrix arrays, the tumor classified with the tumors samples rather than the donor samples. Although this is clearly no more than an anecdotal event, it is an interesting finding.

Though microarray technology represents a major advance and provides a powerful tool for high-throughput expression analysis, the most effective use of this technology requires careful consideration of baseline normal tissue. Our results here emphasize the need for careful examination of what constitutes normal tissue and the importance of future studies to fully characterize normal appearing tissue adjacent to prostate cancers.

## Conclusions

Prostate tumor tissue, histologically normal tissue adjacent to tumors and donor normal prostate tissue exhibit unique gene expression profiles with tumor and adjacent normal profiles more similar to each other than to the donors. These results suggest that normal appearing tissue around prostate tumors may also be undergoing tumor related changes and that careful characterization of these different tissues is necessary to understand molecular changes in leading up to prostate cancer.

## Competing interests

The author(s) declare that they have no competing interests.

## Authors' contributions

URC was involved in the data analysis and interpretation and manuscript writing and revision. RD, as director of the Tissue Bank was involved in obtaining IRB approval and providing deidentified tissue for this research study. CM was involved in data analysis and interpretation. GM and MB as principal investigator and co-investigators of the grant titled "the Molecular Reclassification of Cancer" were responsible for design of the study, providing intellectual input and final approval of the version submitted for review. JG was responsible for providing intellectual direction, guidance for the analysis team, review and final approval of the manuscript.

## Pre-publication history

The pre-publication history for this paper can be accessed here:



## Supplementary Material

Additional File 1 **The fifty most up regulated and down regulated genes from the tumor v donor, tumor v adjacent normal and adjacent normal v donor comparison**. The Affymetrix probe set id, gene names and assignment of biological process for each gene are shown. The biological process annotation includes information from Affymetrix, gene ontology and literature references. Click here for file

## Figures and Tables

**Table 1:** Differential gene expression in the tumor v donor, tumor v adjacent normal and adjacent normal v donor comparisons. The number of genes identified as differentially regulated at each delta (observed d value – expected d value; for details, see Materials and Methods) are shown. Also, shown are the number of up and down regulated genes at each delta. For each of these deltas, the false discovery rate was no greater than 2.5%.

	**Delta 3.0**	**Delta 2.0**	**Delta 1.56**
**Tumor v Donor**	65	474	928
Up Regulated	10	121	305
Down Regulated	55	353	623

**Tumor v Adjacent Normal**	0	92	382
Up Regulated		0	58
Down Regulated		92	324

**Adjacent Normal v Donor**	12	86	254
Up Regulated	5	25	63
Down Regulated	7	61	191
